# Toxicity evaluation of sulfamides and coumarins that efficiently inhibit human carbonic anhydrases

**DOI:** 10.1080/14756366.2020.1822829

**Published:** 2020-09-17

**Authors:** Ashok Aspatwar, Emanuela Berrino, Silvia Bua, Fabrizio Carta, Clemente Capasso, Seppo Parkkila, Claudiu T. Supuran

**Affiliations:** a Faculty of Medicine and Health Technology, Tampere University, Tampere, Finland; b Neurofarba Department, Sezione di Chimica Farmaceutica e Nutraceutica, Università degli Studi di Firenze, Firenze, Italy; ^c^ Institute of Biosciences and Bioresources, CNR, Napoli, Italy; d Fimlab Ltd., Tampere, Finland; e Tampere University Hospital, Tampere, Finland

**Keywords:** Carbonic anhydrases, inhibitors, toxicity screening, zebrafish

## Abstract

Here, we report a toxicity study, conducted on zebrafish larvae, of a series of coumarin and sulfamide compounds that were previously reported as inhibitors of human (h) metalloenzymes, carbonic anhydrases (CAs, EC 4.2.1.1). Due to the high relevance of hCA inhibitors as theragnostic agents, it is of pivotal importance to address safety issues that may arise from the initial *in vivo* toxicological assessment using zebrafish, a relevant model for biomedical research. None of the reported compounds showed adverse phenotypic effects or tissue damage on developing zebrafish larvae after 5 days of exposure. Our study suggests that the coumarin and sulfamide derivatives considered here are safe and suitable for further development and testing.

## Introduction

Early efforts in medicinal chemistry to discover lead compounds and improve their potency often occur independently from a proper evaluation of toxicity issues^1^. As a result, it is estimated that only one out of nine potential new drugs successfully passes through clinical development and complies with the required safety regulations^2^. According to the Food and Drug Administration (FDA), it is estimated that a 10% improvement in predicting drug safety-related failures before entering clinical trials would save up to 100 million USD in developmental costs per drug^3^. In this context, many efforts have been made to involve toxicological evaluations in the early process of drug development^4–5^.

A commonly used preclinical safety assessment is based on cellular assays, which are limited to drug-induced cell-autonomous effects. Such data need to be integrated with the data obtained from a large-scale toxicity evaluation, which considers toxicological effects within a complex organism. Although higher vertebrate models are considered necessary for detailed preclinical characterisation of drug candidates, they are adopted only when strictly required since they are associated with both ethical concerns and higher costs. Therefore, pharmacologists must consider other *in vivo* models that offer quick, non-cumbersome and cost-effective platforms for early-stage toxicity screening^6^. Of particular interest are *Danio rerio* (zebrafish) vertebrate models, which have become a widely accepted tool in preclinical drug discovery^7^. More specifically, embryonic and larval zebrafish allow for rapid *in vivo* tests and developmental toxicity assays of chemical compounds^8–14^. In addition, the toxicity profiles of chemical compounds in zebrafish have been shown to be relatively consistent with human studies^3^^,^^8^^,^^9^^,^^15^. Other advantages include their small dimensions, cost-effectiveness and easy breeding in large numbers, as a single zebrafish pair can produce approximately 200 embryos per year. The embryos/larvae measure approximately 1–4 mm long and can live up to a few days without external feeding^16^. For the safety assessment, the embryos can be maintained in 24-well plates with 1 ml of embryonic medium containing the chemical compound to be examined^11^. The embryos/larvae easily absorb small molecules through their skin and gills, and the compounds can enter the body orally when the larvae begin to swallow at 3 days post-fertilisation (dpf). The embryos develop rapidly *ex utero*, with most organs becoming fully functional at 2–5 dpf^11^. In addition, the transparency of the larvae allows *in vivo* examination using a stereomicroscope. No special permission is required for the use of 1–5 dpf larvae because they are considered primitive organisms that survive without external feeding^11^^,^^17^. Zebrafish models are tolerant to low concentrations of dimethyl sulfoxide (DMSO) (a widely used vehicle for many chemical compounds^18^) and require very small amounts of drugs to carry out the tests^19–21^.

**Figure 1.**
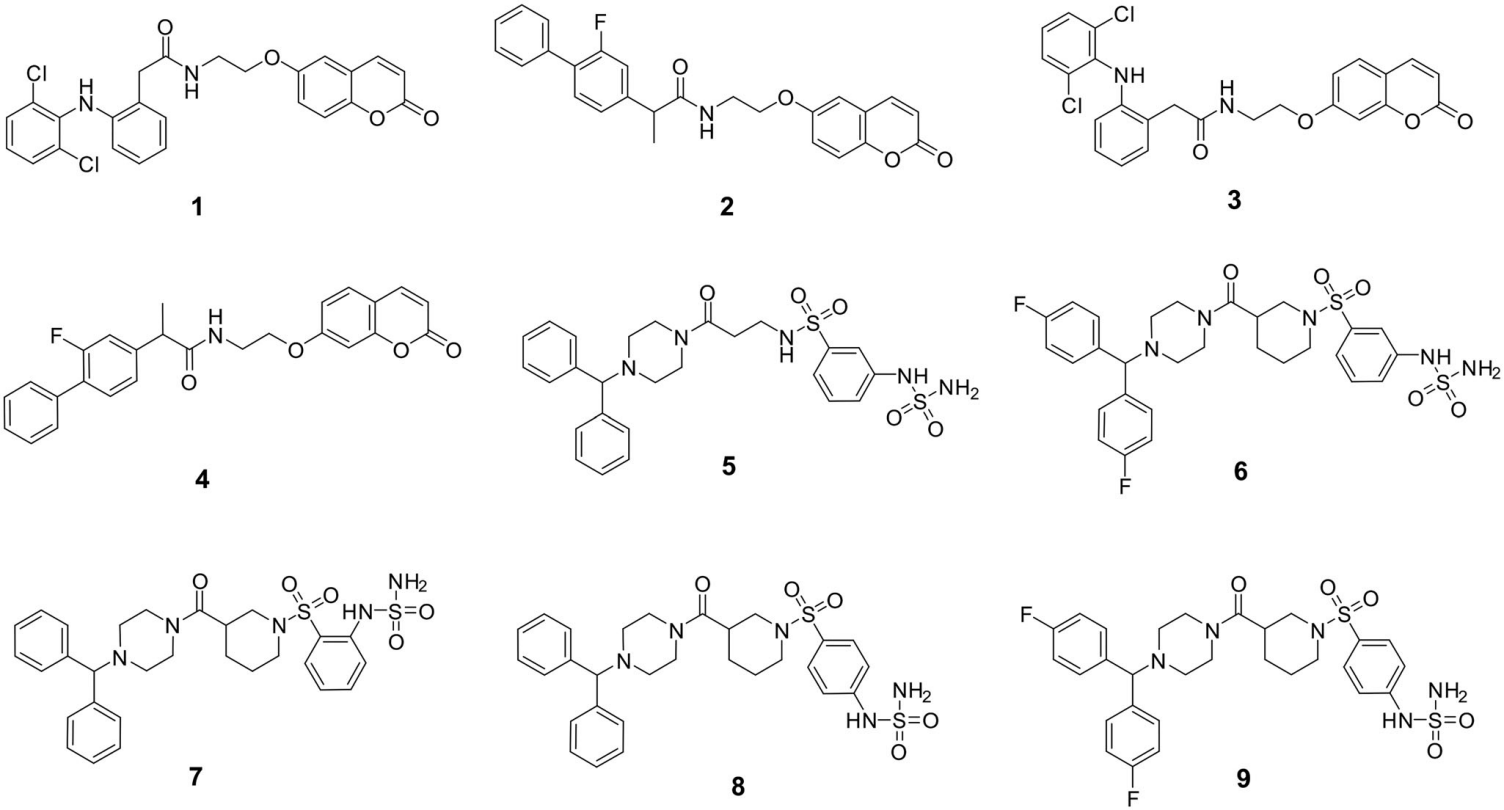
Chemical structures of compounds **1–9**^26^^,^^28^.

**Figure 2.**
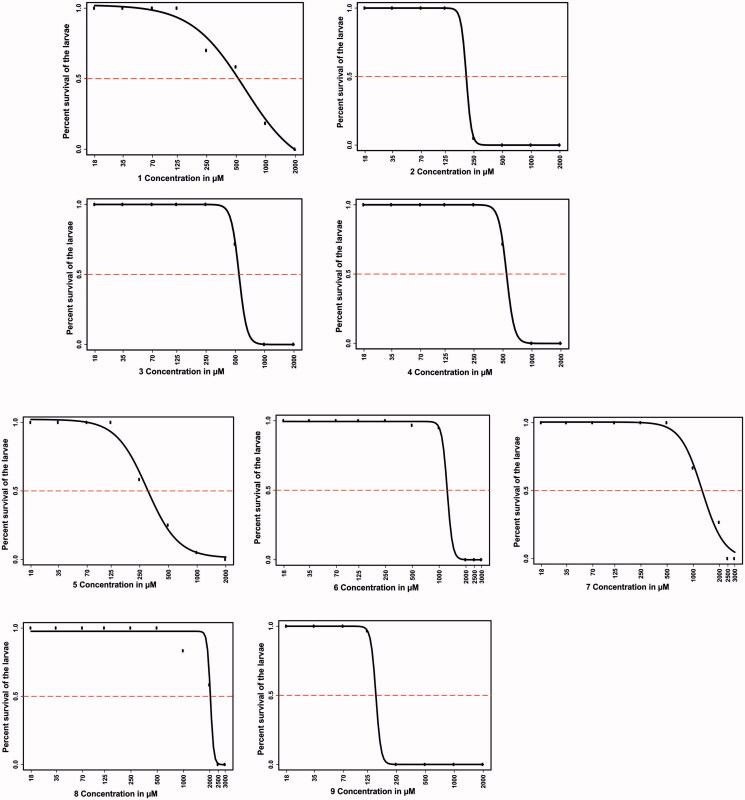
The LC_50_ values of the tested compounds. The upper part of the figure (compounds **1**–**4**) shows the LC_50_ values for the four coumarins, and the lower part (compounds **5**–**9**) shows the LC_50_ values for the sulfamides. The LC_50_ doses for the compounds were determined based on 50% mortality of the larvae at the end of 5 days after exposure of the embryos to different concentrations of any tested compound. LC_50_ doses were determined after three independent experiments with similar experimental conditions were performed (for each compound, *n* = 90).

In the present study, we report a toxicity evaluation of sulfamide- and coumarin-type compounds in zebrafish. Some of us previously reported these compounds to be effective inhibitors of human (h) carbonic anhydrase (CA; EC 4.2.1.1) metalloenzymes. In particular, they include (1) the commercially available nonsteroidal-anti-inflammatory drugs (NSAIDs) diclofenac and flurbiprofen linked to the 6- and 7-positions to the coumarin scaffold (i.e. compounds **1–4** in Figure 1) and (2) benzhydryl piperazine sulfamide-containing derivatives **5–9** (Figure 1).

Our interest in such structures is mainly because coumarins and sulfamides are widely used chemical moieties included in drugs having poly-pharmacological targets, including hCAs^22–27^. In this context, our study aims to report a preliminary toxicity evaluation focussed on such moieties.

## Materials and methods

### Chemistry

Compounds **1–9** considered in this manuscript were synthesised according to procedures previously reported^26^^,^^28^^,^^29^. They were all properly characterised by ^1^H-NMR, ^13 ^C-NMR and mass spectra analysis^26^^,^^28^^,^^29^.

### *In vitro* carbonic anhydrase inhibition assay

The CA-catalysed CO_2_ hydration activity was performed on an Applied Photophysics stopped-flow instrument using phenol red at a concentration of 0.2 mM as a pH indicator with 20 mM HEPES (pH 7.5) as the buffer and 20 mM Na_2_SO_4_. The initial rates of the CA-catalysed CO_2_ hydration reaction were followed for a period of 10–100 s at the maximum absorbance of 557 nm^30^. The CO_2_ concentrations ranged from 1.7 to 17 mM. For each inhibitor, six traces of the initial 5–10% of the reaction were used in order to determine the initial velocity. The uncatalysed reaction rates were determined in the same manner and subtracted from the total observed rates. Stock solutions of inhibitor (0.1 mM) were prepared in distilled water, and dilutions to 0.01 nM were prepared. Solutions containing inhibitor and enzyme were preincubated for 15 min at room temperature (for sulfamide-based compounds) and 6 h (for coumarin-based compounds) prior to the assay in order to allow the formation of the enzyme-inhibitor complex. Inhibition constants were determined with nonlinear least-squares protocols using PRISM 3, as reported earlier^26^^,^^28^^,^^29^, and were the mean from at least three different measurements. All hCAs represented recombinant proteins that were obtained in-house as reported earlier^26^^,^^28^^,^^29^.

### Toxicity evaluation

#### Inhibitors

Compounds were dissolved in DMSO (Sigma-Aldrich, St. Louis, MO) to prepare 100 mM stock solutions. Before the start of each experiment, a series of dilutions were made from the above stock in the embryonic medium [5.0 mM NaCl, 0.17 mM KCl, 0.33 mM CaCl_2_, 0.33 mM MgSO_4_, and 0.1% w/v methylene blue (Sigma-Aldrich)]. Embryos at 1-day post fertilisation (dpf) were exposed to each of the diluted inhibitor solutions.

#### Maintenance of the zebrafish

AB strains of wild-type adult zebrafish were maintained in an incubator at 28.5 °C^10^. To collect embryos, 3–5 pairs of male and female fish were set up overnight for breeding^11^. The next morning, 1–2 h post fertilisation (hpf) embryos were collected from the overnight breeding tanks in a sieve and rinsed with embryonic medium [5.0 mM NaCl, 0.17 mM KCl, 0.33 mM CaCl_2_, 0.33 mM MgSO_4_, and 0.1% w/v methylene blue (Sigma-Aldrich)]. The collected embryos were maintained in an incubator at 28.5 °C overnight. The inhibitor toxicity evaluation studies were performed using fish that were 24 hpf. All zebrafish experiments were performed at the zebrafish core facility, Tampere University, Finland, according to the protocol used in our laboratory^11^.

#### Ethics statement

Tampere University has an authorised zebrafish core facility with permission granted by the National Animal Experiment Board (ESAVI/7975/04.10.05/2016). Experiments using developing zebrafish embryos were performed according to the Provincial Government of Eastern Finland Province Social and Health Department Tampere Regional Service Unit protocol # LSLH-2007–7254/Ym-23. Care was taken to ameliorate suffering by euthanising the 5 dpf larvae by prolonged immersion in a petri dish containing an overdose of tricaine (Sigma-Aldrich) before fixing in buffered formaldehyde for histochemical analysis.

#### Determination of LC_50_

The LC_50_ values of the inhibitors were determined using 1-dpf embryos at 10–12 different concentrations. For each inhibitor concentration, we used 30 1-dpf larvae^11^^,^^31^. In each group, the larvae were exposed to different inhibitor concentrations ranging from 12.5 μM to 3 mM. The dose-response curve (DRC) was calculated using DRM of the DRC R package^32^. The control groups constituted an equal number of untreated larvae and larvae treated with 1% DMSO. Toxicological evaluation studies were performed in 24-well plates (Corning V R Co-star V R cell culture plates). In each well, we placed 1–2 1-dpf embryos in 1 ml of embryonic medium containing a diluted inhibitor. A minimum of three sets of experiments was carried out for each inhibitor. The mortality of the larvae was checked every 24 h until 5 days after exposure to the inhibitors.

#### Phenotypic analysis of control and inhibitor-treated larvae

We assessed the effects of the inhibitors on zebrafish larvae after 5 days of exposure to the drugs by analysing eight observable phenotypic parameters: (1) hatching, (2) oedema, (3) movement pattern, (4) yolk sack utilisation, (5) heartbeat, (6) body shape, (7) swim bladder development and (8) otolith sac development using a stereomicroscope, recording the observations for each group. Images of the developing larvae were taken using a stereomicroscope attached to a camera described in our standard protocol for the assessment of toxicity and safety of the chemical compounds^11^.

#### Swim pattern analysis

The movement of the zebrafish larvae was tracked from day 4 of exposure to these inhibitors. Similarly, a detailed analysis of swim pattern was performed at the end of the 5th day after exposure to the inhibitors. For movement pattern analysis, approximately 10–15 zebrafish larvae were placed in a 35 mm × 15 mm petri dish containing embryonic medium, and the larvae were allowed to settle in the petri dish for 1 min. The movement of the zebrafish larvae was then observed under the microscope for 1 min. A 1-min video was taken for each group of larvae that were treated with a 125 μM concentration of inhibitor. The swim patterns were compared with those of the control group zebrafish larvae that were not treated with an inhibitor.

#### Histological studies

Histological analyses were performed to analyse the effects of inhibitors on the internal tissues of the larvae that were treated with different concentrations of the tested inhibitors, the control group larvae that were not treated with an inhibitor and the larvae treated with 1% DMSO. After 5 days of exposure to different inhibitor concentrations, the larvae were washed with phosphate-buffered saline (PBS) immersed in excess amounts of tricaine to anaesthetise the larvae. The prepared larvae were transferred to a 1.5 ml microcentrifuge tube and fixed in buffered formaldehyde (formaldehyde solution 4%, pH 6.9) in PBS for 3 h at room temperature or overnight at 4 °C. After fixation, the larvae were transferred to 70% ethanol and stored at 4 °C before embedding in paraffin. The paraffin-embedded samples were sectioned into 5 μm thick slices for routine histochemical haematoxylin-eosin (H&E) staining. The slides containing the stained tissue sections were examined and photographed using a Nikon Microphot microscope (Nikon Microphot-FXA, Japan). All procedures were carried out at room temperature unless stated otherwise.

#### Statistical analysis

GraphPad Prism software (5.02) was used to perform statistical analysis. For statistical analysis of the toxicity parameters, a two-tailed Fisher’s test was used, and *p*-values below 0.05 were considered significant.

## Results and discussion

### Chemistry

Compounds **1–9** were synthesised according to procedures reported in the literature^26^^,^^28^ and characterised by ^1^H-NMR, ^13 ^C-NMR and mass spectra (Figure 1).

### Carbonic anhydrase inhibition

All compounds reported in the current manuscript were tested *in vitro* for their inhibitory activity against several hCAs (I, II, IV, VII, IX and XII), and the corresponding data are reported in Table 1^26^^,^^28^–^29^.

**Table 1.** Inhibition data of human CA isoforms hCA I, II, IV, VII, IX, and XII with the derivatives reported here and the standard sulphonamide inhibitor acetazolamide (**AAZ**) by a stopped-flow CO_2_ hydrase assay^30^.

K_I_ (nM)*						
	hCA I	hCA II	hCA IV	hCA VII	hCA IX	hCA XII
1	>10,000	>10,000	5.6	>10,000	28.9	92.6
2	>10,000	>10,000	0.81	>10,000	23.5	5.9
3	>10,000	>10,000	9.3	>10,000	89.7	80.9
4	>10,000	>10,000	8.8	>10,000	159.4	>10,000
5	83.1	418.6	2359.9	N.D.	1024.1	N.D.
6	45.8	753.4	1382.2	N.D.	296.5	N.D.
7	604.6	89.8	314.0	N.D.	>10000	N.D.
8	153.2	455.2	364.4	N.D.	1410.8	N.D.
9	326.1	786.0	466.6	N.D.	902.3	N.D.
AAZ	250.0	12.0	74.0	2.5	25.0	5.7

*Mean from 3 different assays by a stopped-flow technique (errors were in the range of ±5–10% of the reported values).

N.D.: not determined.

Structure-activity relationship (SAR) analysis of the tested compounds is reported below:

1. Coumarin-containing CAIs **1–4** showed to be ineffective inhibitors of the cytosolic hCAs I, II and VII at the concentration range considered (K_Is_ > 10000 nM). Strong inhibition against the remaining isoforms hCA IV, IX and XII was observed (Table 1), with the only exception represented by compound **4**, which showed to be ineffective against hCA XII. In general, the preferential inhibition of hCA IV over the tumour-associated isoforms IX and XII can be noticed within the series (Table 1). The 6-substituted coumarin compound **2**, containing flurbiprofen as NSAID moiety, was particularly effective in inhibiting hCA IV at subnanomolar K_I_ value (i.e. 0.81). Interestingly, its 7-coumarin regioisomer **4** resulted in 10.9-fold less potent on the same enzymatic isoform (K_I_s of 0.81 and 8.8 nM for compound **2** and **4,** respectively). The inhibition data also showed the 6-substituted coumarin regioisomers **1** and **2** being far more potent inhibitors of hCAs IV and IX when compared to their 7-counterparts. As for hCA XII, such a trend was observed only for coumarin regioisomers containing flurbiprofen **2** and **4** (5.9 nM and >10000 nM for the 6- and 7-regioisomers, respectively), whereas, for the diclofenac containing compounds **1** and **3**, 7-isomer **3** showed to be 1.14 fold more potent than the 6-regioisomer **1** (Table 1).
2. As for the benzenesulfonamide bearing compounds **5-8,** their kinetic profiling allowed us to evaluate the impacts of small structural modifications on hCAs I, II, IV and IX inhibition potencies. The starting compound **5**, bearing a β-alanine spacer between the benzenesulfonamide head and benzhydryl piperazine tail, showed medium inhibition potency against hCA I (K_I_ 83.1 nM), whereas the remaining isoforms considered for this study (hCA II, IV and IX) were inhibited at higher concentrations values, spanning between 418.6 and 2359.9 nM. Conformational restriction of the ethyl linker in **5** by means of a nipecotic acid spacer, along with the substitution of aromatic hydrogens with fluorine^33^ afforded the compound **6**. Interestingly, compound **6** showed almost twice the inhibition potency against hCA I and IV (i.e. 1.8 and 1.7-fold, respectively) when compared to the more flexible analogue **5**. As for the tumour-associated hCA IX, a relevant increase of the potency up to 3.5-fold was also observed. The opposite effect was registered against hCA II, as the loss of flexibility going from **5** to **6** determined a 1.8-fold decrease of the inhibitory potency against this isoform (see Table 1). Application of the same conformational restriction to **5** and switching of the sulfamide moiety from *meta* to *ortho* position afforded the derivative **7**. Such a modification spoiled the inhibition potency against hCA I and IX (K_I_s of 604.6 and >10,000 nM, respectively) but resulted in its enhancement against hCA II and IV isoforms (Table 1). Assuming the *ortho*-substituted compound **7** as a new reference compound in our SAR investigations, we also decided to evaluate the *in vitro* inhibition potency of a compound in which the sulfamide moiety was placed in *para* position of the aromatic ring. When compared to **7**, compound **8** was more effective in inhibiting hCA I (K_I_s of 604.6 and 153.2 nM, respectively). Moreover, this structural modification restored the activity against the tumour-associated hCA IX (K_I_ 1410.8 nM). Conversely, compound **8** was 5.1-fold less effective in inhibiting hCA II (K_I_s of 89.8 and 455.2 nM, respectively), whereas the inhibition potency against hCA IV was only slightly affected (K_I_s 314.0 and 364.4 nM for **7** and **8,** respectively). In light of our interest in the effects of the fluorine in medicinal chemistry, such halogen atoms were introduced at the benzhydryl rings in **8** to afford the compound **9**^10^^,^^33^. This modification was detrimental for the inhibition potencies against hCA I, II and IV isoforms (see Table 1). Conversely, a clear enhancement of the inhibition potency against the tumour-associated isoform hCA IX was observed, with compound **9** being 1.56-fold more potent when compared to the non-fluorinated analogue **8** against this isoform. This result is in agreement with what observed previously going from compound **5** to **6**. Overall, the kinetic data on the investigated compounds allowed us to decipher the effects of regioisomeric manipulation, conformational restriction and halogen replacement on both *in vitro* enzymatic activity of hCAs and isoform selectivity. Such effects were particularly evident since the molecules considered are of small dimensions and thus allowed to extract clean and immediate structural data, which will be of pivotal importance for the future development of molecular scaffolds of pharmaceutical interest within the field. In particular, the contemporary presence of fluorine atoms on the benzhydryl piperazine tail connected to the *meta*- substituted benzenesulfonamide head by means of a quite constrained nipecotic spacer, resulted to be particularly favourable to the hCA IX inhibition (i.e. compound **6**). On the other hand, non-fluorinated *ortho*- and *para*-substituted analogues **7** and **8** showed to be more potent hCA IV inhibitors when compared to hCA IX. As for the presence of a more flexible β-alanine spacer (i.e. compound **5**), it resulted to confer more selectivity against the cytosolic isoforms hCA I and II over the membrane-associated hCA IV and IX.

### Evaluation of the safety and toxicity of the coumarin and sulfamide derivatives

#### Determination of the inhibitor half-maximal lethal concentration (LC50)

The lethal effects of all compounds on the developing zebrafish embryos exposed to the inhibitors for 5 days were concentration-dependent (Figure 2). Compounds **6**, **7**, and **8** exhibited less than 50% mortality at a concentration up to 1 mM at the end of 5 days of exposure. Significant lethality for compounds **6** and **7** was observed at concentrations higher than 1 mM, while compound **8** reached the half-maximal lethality level only at concentrations above 2 mM, suggesting that they are the least toxic among the evaluated compounds. LC_50_ values for compounds **3** and **4** (500 µM) and for compounds **1**, **2**, **5** and **9** were the lowest (>125 µM). The half-maximal lethal concentrations of the tested inhibitors were higher compared to other similar toxicity and safety assessment studies that we performed earlier using other compounds^10^^,^^14^^,^^34^.

#### Phenotypic analyses of zebrafish larvae treated with inhibitors

**Figure 3.**
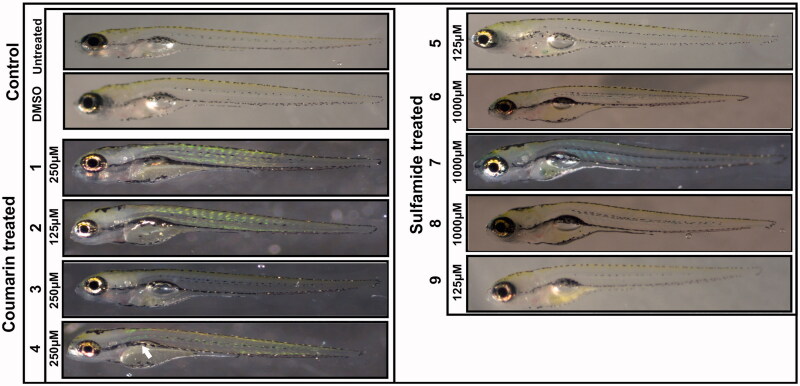
Images of zebrafish larvae in the control and coumarin/sulfamide derivative-treated groups. Representative images of 5 dpf zebrafish larvae exposed to different concentrations of CAIs **1–9** that are considered safe (below the LC_50_). No morphological changes were observed except for with compound **4**, which showed a defect in swim bladder development (white arrow). Images of the control group larvae (not treated with inhibitor) and 1% DMSO-treated larvae showed normal development.

To assess the toxic effects of the inhibitors on developing zebrafish larvae, we analysed eight observable phenotypic parameters, namely, hatching, heartbeat, otolith sac development, yolk sac utilisation, movement pattern, body shape, swim bladder development, and oedema. The parameters were monitored using a stereomicroscope, and observations were recorded for each group compared with control embryos that were not treated with any compound or treated with 1% DMSO. The images in Figure 3 show representative larvae exposed to the compounds with concentrations that are considered safe compared to the control group larvae. The microscopic examination showed no observable changes in the developing zebrafish larvae at concentrations considered safe except for compound **4**, which showed an effect on swim bladder development, and the larvae showed normal phenotypes throughout embryonic development from 2 to 5 dpf.

We further assessed the toxicity of the compounds in zebrafish larvae across the concentration range of 18 μM to 2 mM. Table 2 shows the effects of the compounds on different phenotypic parameters in the 5 dpf zebrafish larvae. The results indicated that none of the inhibitors showed substantial toxic effects below the LC_50_ concentration range. Previous toxicity studies on hCA IX and mycobacterial β-CA inhibitors showed significant effects on larval mortality and observable phenotypic parameters in the concentration range of 50 μM to 300 μM^14^^,^^34^. Therefore, the results of the current study show that both coumarin and sulfamide derivatives exhibit minimal toxicity in the zebrafish larval model.

**Table 2.** Effects of CA inhibitors on the phenotypic parameters of 5 dpf zebrafish larvae^a^.

Cmp^b^	Hatching		Oedema	Swim	H. beat	Y. sac	O. sac	B. shape	S. bladder
1 (250 µM)		100	0	0	0	0	0	0	0
2 (125 µM)		100	0	0	0	0	0	0	0
3 (250 µM)		100	0	0	8	0	0	0	0
4 (250 µM)		100	0	0	0	0	0	3	8
5 (125 µM)		100	0	0	0	0	0	0	0
6 (1000 µM)		100	0	0	0	0	0	0	0
7 (1000 µM)		100	0	0	0	0	0	0	0
8 (1000 µM)		100	0	0	0	0	0	0	0
9 (125 µM)		100	0	0	3	3	0	3	0

^a^The values are in percent. ^b^The figures in the parentheses indicate the concentration of the compound (Cmp).

H. beat: heartbeat; Y. sac: yolk sac; O. sac: otolith sac; S. bladder: swim bladder.

#### Histochemical analysis

**Figure 4.**
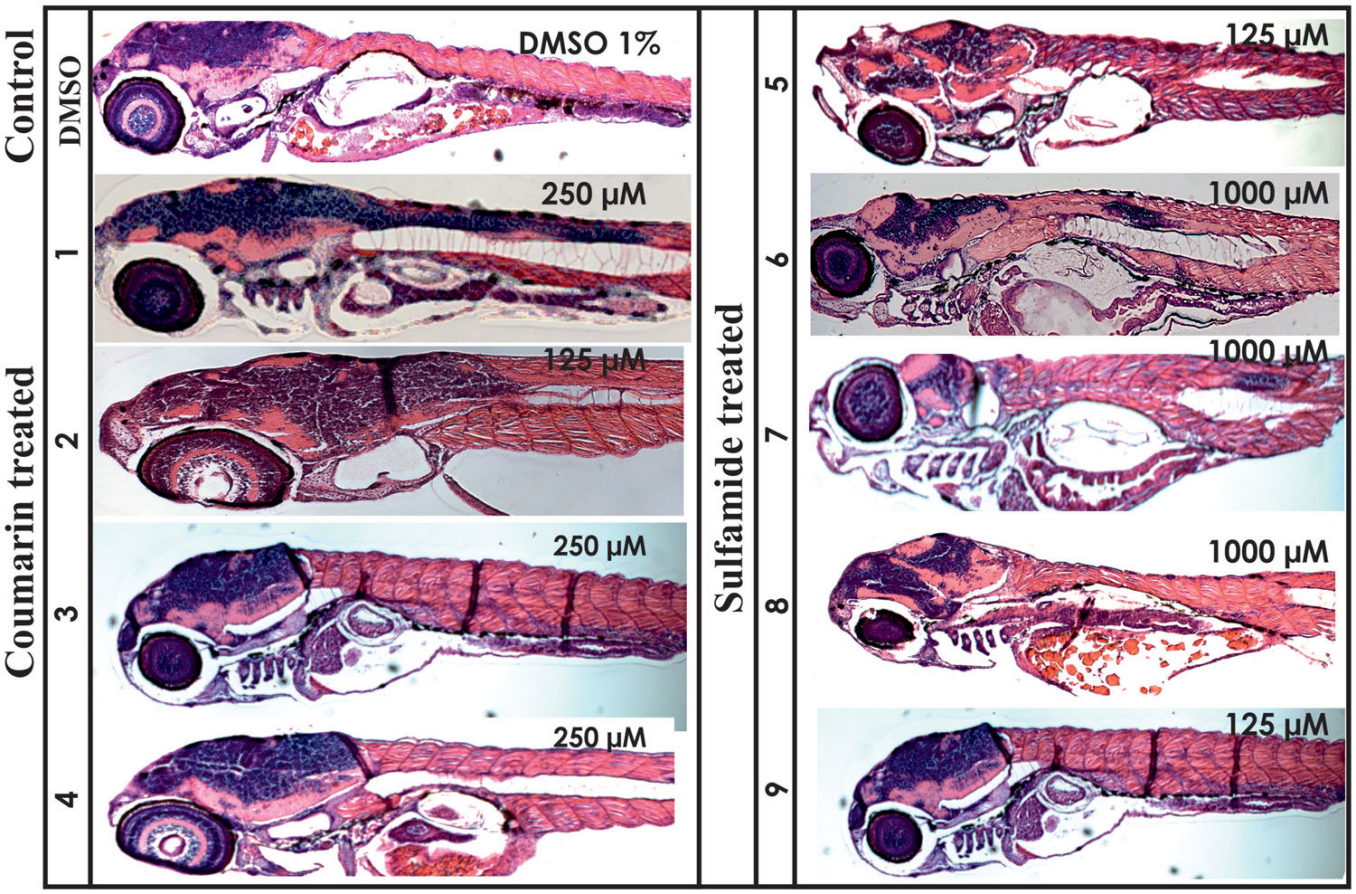
Histochemical analysis of coumarin- and sulfamide-treated 5 dpf zebrafish larvae. The images presented here are from the larvae treated with concentrations that showed no phenotypic defects (the concentration is shown in the top right corner of each image). The images presented here were selected from three independent groups of experiments. The H&E stained sections show no histomorphological abnormalities in the tissues of zebrafish larvae at the end of 5 days of exposure.

The histomorphological effects of different compounds were studied by analysing the 5 dpf zebrafish larvae sections stained with haematoxylin and eosin (H&E). The stained sagittal sections of both the inhibitor-treated and control larvae were compared by light microscopy (Figure 4). The histological examination revealed no adverse effects from the compounds on the tissues of developing zebrafish larvae. Even concentrations up to 2 mM showed no apparent damage to internal tissues, suggesting that these compounds are safe and can be further developed as potential CA-specific inhibitors for preclinical testing in mammals.

#### Analysis of swim patterns

During development, zebrafish embryos are easily affected by chemical compounds compared to adult zebrafish or other animal or cell models. Therefore, they are suitable for detecting subtle toxic effects of chemicals^11^^,^^34^. We, therefore, assessed the potential effects of the coumarin and sulfamide derivatives by studying the swim pattern of the larvae under the microscope at the end of the 5-day compound exposure period. The swim pattern analysis showed no abnormal or ataxic movements from the larvae exposed to concentrations below the LC_50_ (data not shown), suggesting that these compounds are not neurotoxic, as seen in our earlier studies^11^. This result also suggested that the reported derivatives can be considered safe for further preclinical characterisation and development as human isoform-specific CA inhibitors.

## Conclusions

In the present study, we report an *in vivo* toxicity evaluation of a small series of coumarin (compounds **1–4**) and sulfamide (**5–9**)-based CAIs on 1–5 dpf zebrafish larvae. The use of zebrafish vertebrate models in preclinical drug discovery is now a widely accepted tool to conduct rapid *in vivo* tests. These animals can be used in developmental toxicity assays of chemical compounds with a high degree of similarity between the drug responses of zebrafish and humans^3^. Evaluated compounds **1–9** were previously reported^26^^,^^28^^,^^29^ and showed different potency and selectivity profiles against the cytosolic hCA I, II and VII isoforms and the membrane-associated isoforms hCA IV, CA IX and XII. In particular, the coumarin derivatives (**1–4**) did not inhibit hCA I, II and VII in the concentration range tested, whereas low nanomolar inhibitory activity against the membrane-associated isoforms IV, IX and XII was observed. In the sulfamide-based compound series **5–8,** bearing a benzhydryl piperazine tail, the observed kinetic profiles allowed us to draw interesting SARs, considering any possible effects on hCA I, II, IV and IX inhibition due to small structural modifications, such as regioisomeric manipulation, conformational restriction and fluorine insertion. Studies on the lethal concentrations revealed that these compounds exhibit minimal toxicity. In particular, sulfamide-based compound **8** was shown to be safe even at millimolar concentrations at the end of 5 days of exposure, being one of the most promising among the series. Moreover, phenotypic and histochemical analyses revealed that, with the exception of compound **4** showing a defect in swim bladder development, the analysed compounds did not induce observable changes in the developing zebrafish larvae, nor did they show any apparent damage to internal tissues. Moreover, no abnormal or ataxic movement patterns in the larvae exposed to compounds **1–9** were observed, allowing us to consider these compounds to be safe and suitable agents for further development as potential leads in drug development processes.
